# Mortality Rates of the U. S. Armies for the Year Ending June, '62

**Published:** 1864-01

**Authors:** J. J. Woodward

**Affiliations:** U. S. A.


### CHRONICLE OF MEDICAL SCIENCE.

*Mortality Rates of the U. S. Armies for the Year ending June, '62.* By Surgeon J. J. Woodward, U. S. A. [Extracts from Report to Surgeon-General U. S. A.]

The general mortality rate of the armies of the United States during the first year of the rebellion was 67.6 per thousand of mean strength, including with deaths from disease those from wounds and injuries. The mortality from disease alone was 50.4 per thousand; that from wounds and injuries of every kind 17.2 per thousand.

In contrast with these results, it may be stated that the average annual mortality from disease alone, in the United States army, during eighteen years of peace, was 24 per thousand. In the United States army, during the Mexican war, 109.8 per thousand. In the British army, during the Crimean war, 232 per thousand. In the British army, during the year 1859, 9 per thousand. It appears, therefore, that although the mortality of the army from disease, during the first year of the present rebellion, was far heavier than that of our own or of the British army in time of peace, it was much less than that of the armies engaged in the Mexican or the Crimean wars.

The following table exhibits the monthly mortality rates of the army from July 1, 1861, to June 30, 1862. The armies have been consolidated for the purpose of comparison into three great divisions. The first consists of the troops operating on the Atlantic coast between the Appalachian range and the sea, and includes the army of the Potomac and the various coast expeditions. The annual mortality from disease alone among these troops was 33.40 per thousand of mean strength.

The second consists of the troops operating in the central basin of the continent, between the Appalachian and the Rocky Mountains; and includes Western Virginia, the armies under Generals Buell, Grant and Pope, the department of Missouri, with the scattered troops in Kansas, Nebraska, New Mexico and the Northwest. The annual mortality from disease alone in this region was 82.19 per thousand.

The third division consists of the troops on the Pacific slope, between the Rocky Mountains and the sea. It includes those serving in northern and southern California, Oregon and Washington territory. The annual mortality rate was 10.76 per thousand.

It will thus be seen that on the Pacific slope the mortality rate was three times less than on the Atlantic coast, while that of the latter region was twice and a half less than that of the troops serving in the central region.

The small amount of mortality on the Pacific coast is worthy of attention. The rate is hardly greater than that attributed by British and New England statisticians to young men of similar ages in private life. This exemption is in part due, there can be no doubt, to the fact that on the Pacific coast our troops found themselves under conditions much more closely approximating those of peace than of war. But the rate is so much less than has ever been known in the whole United States army in time of peace, that an idea of the superior healthfulness of the Pacific coast is at once suggested. The greater mortality of the central region, as compared with the Atlantic coast, would appear to hold a close relationship to the great prevalence of malarious disease in the valleys of the Mississippi and its tributaries, which is indicated by tables III, IV and V, showing the monthly rates of camp fever, of intermittent fever, and of diarrhœa.

The three great regions above contrasted differ not only in their annual mortality rate, but the relations of mortality to season are also quite different.

Thus, on the Atlantic coast the mortality, after falling off in September, steadily increased during October, November and December, diminished through January and February, and then steadily increased again through March, April, May and June.

In the central region the mortality rates became gradually greater from July, 1861, to March, 1862, diminished in April, increased again in May, and diminished in June.

On the Pacific coast a much more fluctuating course was pursued, and quite unlike either of the others, as will be shown in the following table:

TABLE I.

*Mortality rates of the armies of the United States during the year ending June 30, 1862, expressed in ratio per thousand of mean strength.*

| | |
|---|---|
| Atlantic | 33.04 |
| Central | 82.19 |
| Pacific | 10.76 |

*General Prevalence of Disease.*

The difference between the three regions above contrasted is not so conspicuous in the general sickness rates as in the mortality, yet the whole number taken sick in the central region was greater than on the Atlantic coast, and in this again greater than on the Pacific. In the first, the number taken on sick report during the year was 3364.14 per thousand of mean strength; in the second, 2748.83, and in the third, 2586.60. It will thus be seen that in each of these regions a large proportion of the troops must have been taken sick several times during the year.

Table II. exhibits the ratio of "taken sick" for each of the three regions. It does not indicate the "constant sickness rate," but the total number taken on sick report during the month. The monthly fluctuations exhibited by this table are, of course, much less instructive than those of individual diseases; they serve, however, to indicate a gradual improvement in the sanitary condition of the army during the war.

It would be exceedingly interesting were it possible to present a table representing the "constant sickness rates" for the same period; but the imperfect data in the Surgeon-General's office, for the first year of the war, do not afford the means for computing such a table in a reliable manner.

TABLE II.

*Sickness rates of the armies of the United States during the year ending June 30, 1862, expressed in ratio per thousand of mean strength.*

| | |
|---|---|
| Atlantic | 239.75 |
| Central | 232.83 |
| Pacific | 193.51 |

This makes the per centage for the year, 2748.83 on the Atlantic border; 3368 14 in the Central region, and 2586.60 on the Pacific coast.

*Camp Fever.*

Under the head of camp fever, all the cases reported to the Surgeon-General's office, as typhus, typhoid, common continued, and remittent fevers, are here concluded. Of these several categories it may well be doubted how far the cases reported as typhus were really of that character. From the details furnished by sanitary reports it appears probable that, with perhaps rare exceptions, what was regarded as typhus was, in fact, of a very different nature; severe typhoid fever, with cerebral complications, and congestive intermittents in scorbutic constitutions being shown, in some cases at least, to have been regarded as typhus. This error was not, however, very widely diffused, the whole number of cases reported as typhus amounting to but a few hundred. As for the cases reported as common continued fever, the vast majority appear to have been different only in degree of severity from those reported as typhoid or remittent. Moreover, while a certain amount of uncomplicated enteric and remittent fever certainly did occur, especially at the commencement of the war, the vast majority of the camp fevers of the army were of a mixed character, exhibiting undoubted enteric phenomena, variously combined with the periodicity and other peculiarities of malarial disease, and still further modified by the tendency of incipient scurvy, which is the ordinary concomitant of camp diet. To indicate this mixed nature, the term typho-malarial fever, which I had the honor to suggest to the department in June, 1862, appears appropriate, and, at the present time, is coming into very general use.

A correct understanding of the nature of these fevers is of the utmost importance, as they play a conspicuous part in the mortality of our armies. During the year under consideration 44.5 per cent. of all the deaths from disease were due to camp fevers.

An examination of table III. shows that the frequency and mortality of camp fever differs considerably in the three great regions. On the Atlantic border the annual ratio of cases was 238 99 per thousand of mean strength, and the ratio of deaths to cases was 71.9 per thousand, or one death to every 13 9 cases. In the central region the annual ratio of cases was 319 94 per thousand, and the ratio of deaths 101.8 per thousand cases, or one in 9 8. On the Pacific coast the annual ratio of cases was only 60.95 per thousand, and the ratio of deaths to cases 45.2, or one in 22.1. The severity of camp fever in these several regions is thus shown to differ as considerably as their frequency.

An inspection of the table at once exhibits the autumnal character of the disease. On the Atlantic coast the monthly number of attacks steadily increased until November, 1861, then as steadily diminished until March, 1862; after which they once more increased in frequency. In the central region the maximum was attained in September, 1861, followed by a gradual diminution till March, and a subsequent increase as on the Atlantic coast. On the Pacific coast, although there is less regularity in the fluctuates, it will be observed that October was the maximum month. The most superficial observer cannot fail to be struck with the similarity between these three waves and those of the intermittent fevers, of whose malarial nature there is no doubt, and which are illustrated in the next table.

TABLE III.

*Rates of camp fever in the armies of the United States during the year ending June 30, 1862, expressed in ratio per thousand of mean strength.*

| | |
|---|---|
| Atlantic | 27.07 |
| Central | 27.64 |
| Pacific | 1.78 |

This makes the per centage for the year 238.99 on the Atlantic border; 319.94 in the Central region, and 60.95 on the Pacific coast.

*Intermittent Fever.*

Intermittent fever, although a very frequent affection, has not been the cause of any great mortality. On the Atlantic border the annual ratio of cases was 195.94 per thousand of mean strength, the rate of deaths to cases 6 0 per thousand, or one to 165.9. In the central region the annual ratio was 375.34, the deaths 5.9 per thousand cases, or one to 170.0. On the Pacific coast the annual ratio was 151.68 per thousand of mean strength and no deaths.

The distinctly autumnal character of the disease is well shown in the following table:

TABLE IV.

*Rates of intermittent fevers in the armies of the United States during the year ending June 30, 1862, expressed in ratio per thousand of mean strength.*

| | |
|---|---|
| Atlantic | 16.88 |
| Central | 26 02 |
| Pacific | 12.46 |

This makes the per centage for the year 195.94 in the Atlantic border; 375 34 in the Central region; and 151.68 on the Pacific coast.

*Diarrhœa and Dysentery.*

Diarrhœa and dysentery caused about one-fourth of all the sickness reported. On the Atlantic border more than half the army suffered, and in the central region the number of cases almost equalled the mean strength. Although not nearly so fatal as camp fever, affections of this class were an important cause of the mortality of our army. In the chronic cases, though most generally called diarrhœa, and not dysentery, the colon was the seat of the chief lesion. The most characteristic post mortem appearance was a thickened, softened condition of the mucous membrane, with pigment deposit and enlargement of the solitary follicles, frequently terminating in ulceration, the ulcers being sometimes punctiform, sometimes extensive, and irregular. In this condition the small intestine frequently participated, more or less, but often presented nothing abnormal.

It appears from table V. that the annual ratio of diarrhœa and dysentery on the Atlantic coast was 640.01 cases per thousand of mean strength.

In the central region 994.77 per thousand, and on the Pacific coast 319 64. The relative mortality was, in the Atlantic region, 2 1 deaths per thousand cases, or one in 483; in the Central, 9.6 per thousand, or one in 103.8; on the Pacific, 0 9 per thousand, or one in 1,159.

TABLE V.

*Rates of diarrhœa and dysentery in the armies of the United States during the year ending June 30, 1862, expressed in ratio per thousand of mean strength.*

| | |
|---|---|
| Atlantic | 87.06 |
| Central | 83 02 |
| Pacific | 30.25 |

This makes the per centage for the year 646.01 on the Atlantic border; 994.77 in the Central region; and 319.64 on the Pacific coast.

*Catarrhal Affections.*

Catarrhal affections of every class were exceedingly common, attacking nearly one-half the forces in the field. The relative frequency in the three regions of the country appears to have been about the same. On the Atlantic border 456.47 per thousand of mean strength. In the Central region 427.2 per thousand, and on the Pacific slope 407.61. In all, the frequency of these affections increased greatly during the winter, and diminished during the warmer months. The maximum month being January for the Atlantic and Central, and February for the Pacific region. A large proportion of the severer catarrhal cases occurred as sequelæ to camp measles. The vast majority of the simple catarrhal cases terminated in recovery, the deaths being one to every 1,127.8 cases on the Atlantic coast; one to every 560.0 cases in the Central region; and no deaths occurring from this cause on the Pacific region. A certain number of these catarrhal cases, however, terminated in pneumonia, and thus a part, at least, of the mortality of catarrhal affections is reported under that head. The annual rates of pneumonia for the three regions were as follows: On the Atlantic coast, 25 7 cases per thousand of mean strength, the deaths being 131.1 per thousand cases, or one death to every 7.6 cases; in the Central region the cases were 64.2 per thousand of mean strength, the deaths 239.2 per thousand, or one to every 4.1; on the Pacific slope the cases were 20.9 per thousand of mean strength, the deaths 13.1 per thousand cases, or one to 76.

TABLE VI.

*Rate of catarrhal affections in the armies of the United States during the year ending June 30, 1862, expressed in the ratio per thousand of mean strength.*

| | |
|---|---|
| Atlantic | 11 33 |
| Central | 11.71 |
| Pacific | 17 25 |

This makes the per centage for the year 456 47 on the Atlantic border; 427.20 in the Central region; and 407 61 on the Pacific coast—*Army and Navy Journal.*

